# Construction and Application of Energy Footprint Model for Digital Twin Workshop Oriented to Low-Carbon Operation

**DOI:** 10.3390/s24113670

**Published:** 2024-06-05

**Authors:** Lei Zhang, Cunbo Zhuang, Ying Tian, Mengqi Yao

**Affiliations:** 1 School of Mechanical Engineering, Tianjin University of Commerce, Tianjin 300134, China; 2 Laboratory of Digital Manufacturing, School of Mechanical Engineering, Beijing Institute of Technology, Beijing 100081, China; 3 School of Mechanical Engineering, Tianjin University, Tianjin 300072, China

**Keywords:** workshop, energy consumption model, digital twin, energy consumption optimization

## Abstract

To address the difficulty of accurately characterizing the fluctuations in equipment energy consumption and the dynamic evolution of whole energy consumption in low-carbon workshops, a low-carbon-operation-oriented construction method of the energy footprint model (EFM) for a digital twin workshop (DTW) is proposed. With a focus on considering the fluctuations in equipment energy consumption and the correlation between multiple pieces of equipment at the workshop production process level (CBMEatWPPL), the EFM of a DTW is obtained to characterize the dynamic evolution of whole energy consumption in the workshop. Taking a production unit as a case, on the one hand, an EFM of the production unit is constructed, which achieved the characterization and visualization of the fluctuations in equipment energy consumption and the dynamic evolution of whole energy consumption in the production unit; on the other hand, based on the EFM, an objective function of workshop energy consumption is established, which is combined with the tool life, robot motion stability, and production time to formulate a multi-objective optimization function. The bee colony algorithm is adopted to solve the multi-objective optimization function, achieving collaborative optimization of cross-equipment process parameters and effectively reducing energy consumption in the production unit. The effectiveness of the proposed method and constructed EFM is demonstrated from the above two aspects.

## 1. Introduction

The energy consumption of the manufacturing industry accounts for approximately 30% of the total global energy consumption and is one of the main drivers of energy consumption growth [1,2]. In response to the growing energy demand and continuous promotion of energy conservation and emission reduction, China made a commitment at the United Nations General Assembly in 2020 to start reducing emissions within the next decade and achieve carbon neutrality by 2060 [3]. Manufacturing enterprises, as the main contributors to energy consumption and carbon emissions, are the focus of future energy conservation and emission reduction, while reducing workshop energy consumption is one of the important means for manufacturing enterprises to save energy and reduce emissions.

Currently, reducing workshop energy consumption is mainly carried out from the aspects of production cycle balancing [4], process planning [5], and production scheduling [6]. The impact of equipment process parameters on workshop energy consumption has not been considered yet. The fundamental reason is that the equipment energy consumption exhibits a time-varying fluctuation due to changes in process parameters (such as machine tool process parameters [7] and robot operating parameters [8]), which is difficult to characterize. This further results in a dynamic evolution of the whole energy consumption in a workshop.

The dynamic evolution of whole energy consumption in a workshop is not only related to the fluctuation in equipment energy consumption but is also closely related to the correlation between multiple pieces of equipment at the workshop production process level (CBMEatWPPL). During workshop operation, the production process and scheduling plan need to be dynamically adjusted, resulting in dynamic changes in the CBMEatWPPL. This poses greater challenges to the accurate characterization of the dynamic evolution of whole energy consumption in workshops.

The existing research mainly utilizes technologies such as the Internet of Things [9] and machine learning [10] to model the energy consumption of workshops and equipment. The characterization of energy consumption has been achieved by fully utilizing the collection and simulation data of workshops and equipment. However, these studies assume that the power remains constant during the equipment operation. The CBMEatWPPL is not considered either. Due to both factors, the dynamic evolution of whole energy consumption in workshops is difficult to accurately characterize.

A digital twin workshop (DTW) can dynamically characterize equipment status, production progress, and product performance, and can provide feedback control and decision-making for physical workshops. It has shown potential in modeling workshop energy consumption [11]. Therefore, a construction method of the energy footprint model (EFM) for a DTW is proposed to accurately characterize the dynamic evolution of whole energy consumption in workshops. Taking a production unit as a case, an EFM for the production unit is established. The energy consumption fluctuation in a single piece of equipment and the dynamic evolution of whole energy consumption in the production unit are characterized. Based on the EFM, an objective function of workshop energy consumption is formulated. With workshop energy consumption as the main objective and tool life, robot motion stability, and production time as secondary objectives, the collaborative optimization of cross-equipment process parameters is carried out to reduce the production unit energy consumption.

The remainder of this paper is organized as follows. Section 2 reviews the relevant literature and presents the research gaps and main contributions of this article. Section 3 introduces the workshop EFM and presents its modeling method in detail. Section 4 implements a case study. Section 5 concludes the paper and gives the future directions.

## 2. Literature Review

### 2.1. Energy Consumption Modeling of Workshop

Before conducting energy consumption modeling, it is necessary to analyze the composition and influences of the energy consumption of workshops or equipment. Zhou et al. divided energy consumption models (ECMs) into three categories, which included a linear type of cutting ECM based on the material removal rate, a detailed parameter type of cutting energy consumption correlation models, and a process-oriented machining ECM. It was pointed out that specific energy consumption, which referred to the mapping relationship between energy consumption and process parameters, reflected the energy efficiency of machine tools [12]. Brossog et al. measured the power of robots under different payloads and tool center velocities, and found out that both the payload and tool center velocity had an impact on the robot power [13]. Yan et al. studied quantitative relationships between operating parameters and energy consumption for industrial robots. It was indicated that the robot energy consumption was related to the displacement, speed, and acceleration of the robot tool center. On this basis, a mapping relationship between robot energy consumption and the above parameters was obtained [14]. It can be seen that understanding the composition and impacts of energy consumption lays the foundation for energy consumption modeling.

In terms of modeling methods for workshop energy consumption, Herrmann et al. proposed a modeling method of an energy-oriented simulation model for planning manufacturing systems. The equipment energy consumption was simulated with operating parameters. The workshop energy consumption was calculated through accumulation [15]. Ayerbe et al. proposed an online data-driven energy modeling approach. The dynamic behavior of workshop energy consumption was modeled by using CNC data and PLC signals [16]. These studies assume that equipment has a constant power and do not consider the impact of changes in workshop operating conditions, making it difficult to achieve accurate characterization of workshop energy consumption. To this end, Li et al. presented a data-driven hybrid Petri-net by combining a state-based energy consumption modeling method with a machine learning algorithm. A meta model of energy consumption behavior for a workshop was established. It was integrated into the digital twin of an energy-efficient manufacturing system, which had higher accuracy in energy behavior prediction [11].

To sum up, most existing methods for analyzing and modeling workshop energy consumption ignore the impact of fluctuations in equipment energy consumption, and do not take into account the dynamic changes in the CBMEatWPPL with the production process. As a result, it is difficult to achieve accurate characterization of the dynamic evolution of whole energy consumption in a workshop.

### 2.2. Energy Consumption Optimization of Workshop

The optimization of workshop energy consumption is usually based on the workshop ECM. Based on a simulation model of workshop energy consumption, the production energy consumption of the workshop was reduced by adjusting the equipment operation time and workshop’s production cycle [4]. Considering the insufficient accuracy of the energy consumption simulation model, Sobottka et al. collected production data to improve the simulation model of workshop energy consumption. Simultaneously considering the objectives of workshop energy consumption, production cost, and production time, the workshop’s production scheduling was optimized, thereby reducing the workshop energy consumption [17].

Compared with the simulation model of workshop energy consumption, a production data-based energy consumption model has higher accuracy and is more conducive to optimizing workshop energy consumption. Wang et al. used an artificial neural network and the production of big data to identify equipment with abnormal energy consumption, and determined the causes of abnormal energy consumption. The corresponding suggestions for these different reasons were provided, thereby achieving the optimization of energy consumption and manufacturing performance in the workshop [18]. Loffredo et al. proposed a reinforcement learning-based workshop energy efficiency control strategy to address the difficulty in acquiring comprehensive information about system dynamics in real-world scenarios. Without relying on full knowledge of the system dynamics, the workshop energy consumption was reduced [19]. Barenji et al. proposed a digital twin-driven approach for energy consumption optimization in workshops, which was combined with agent-based decision-making for the real-time optimization of motion planning. By integrating, analyzing, and optimizing physical and virtual workshops, the workshop energy consumption was reduced [20]. Regarding the issue of non-real-time optimization of workshop energy consumption, Xia et al. proposed a real-time optimization method for workshop energy consumption based on digital twin technology. By establishing a workshop digital twin model and combining the energy consumption characteristics, unit production time, production state, and behaviors of the equipment, a real-time energy consumption optimization considering fault interference was developed and validated in a shell production line [21]. On the basis of a digital twin-enabled energy-saving platform, Zhang et al. proposed a stochastic dynamics model via max-plus algebra to characterize the spatio-temporal nature of state transition. Then, a model predictive control approach for energy consumption optimization was provided to determine the optimal start–stop control signal [22]. These above studies have all upgraded the effectiveness and real time of energy consumption optimization by improving the accuracy of models of workshop energy consumption. But, the impact of equipment process parameters on workshop energy consumption is ignored.

It should be noticed that current studies on the energy consumption optimization of workshops mainly focus on production cycle balancing, process planning, and production scheduling. With the support of big data, reinforcement learning, and digital twin, the energy consumption optimization of workshops has been further improved. However, these studies have not yet considered the influences of cross-equipment process parameters on the whole energy consumption in workshops, resulting in deviations in the optimization results and poor optimization potential.

### 2.3. Research Gaps and Contribution

According to the above literature review, some research gaps can be found. First, the existing workshop ECM assumes that the power remains constant during the equipment operation. The fluctuation in equipment energy consumption and the CBMEatWPPL have not been considered. Second, the current research on energy consumption optimization in workshops mainly focuses on production cycle balancing, process planning, and production scheduling, rather than cross-equipment process parameters. These research gaps highlight the need for advancements in the modeling and optimization of workshop energy consumption.

Aiming at overcoming these research limitations, a construction method of an EFM for a DTW towards low-carbon operation is proposed. Taking a production unit as a case, an EFM for the production unit is established. Based on the EFM, the cross-equipment process parameters are collaboratively optimized to reduce the energy consumption of the production unit. The main contributions of this paper include the following:

(1) Construction method of an EFM for a DTW. With a focus on considering the impact of fluctuations in equipment energy consumption, an ECM for a single piece of equipment is established. On this basis, considering the CBMEatWPPL, a whole energy consumption model for a workshop is constructed. By integrating the spatial geometric model, operational logic model, ECM, and data interaction model of the workshop, the EFM of a DTW is obtained, achieving accurate characterization of the dynamic evolution of whole energy consumption in a workshop.
(2) Collaborative optimization of cross-equipment process parameters based on an EFM. Based on the EFM, an objective function of workshop energy consumption is established. With workshop energy consumption as the main objective and tool life, robot motion stability, and production time as secondary objectives, the cross-equipment process parameters are collaboratively optimized using the bee colony algorithm. The energy consumption of the production unit is reduced from the aspect of equipment process parameters.

## 3. Construction of EFM for DTW

### 3.1. Architecture of EFM for DTW

The workshop production process is accompanied by the flow of materials and energy, among which the energy flow mainly involves the electrical energy consumption of various pieces of equipment. Analyzing the composition and flow of energy consumption in workshops helps establish a quantitative flow model for energy storage, transmission, conversion, and consumption in workshops. This model is defined as the EFM of workshops, which is inspired by the energy footprint graphical model of products [23].

**Figure 1:**
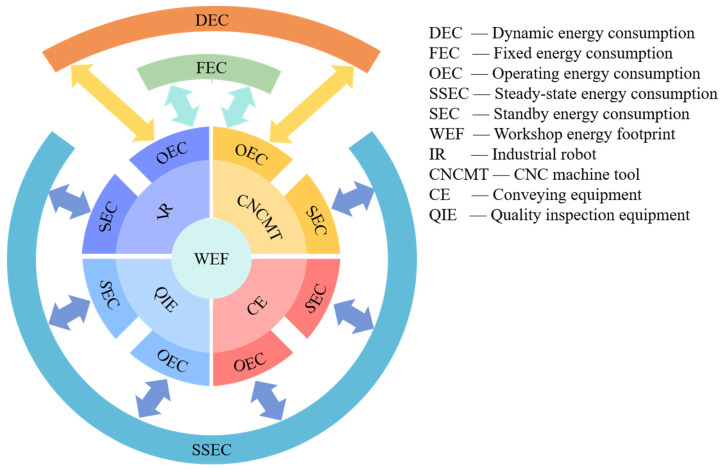
Energy footprint composition of workshops.

Typical workshops in the mechanical manufacturing industry are mainly composed of CNC machine tools, industrial robots, quality inspection equipment, and conveying equipment. The energy footprint composition of workshops is shown in Figure 1. According to the equipment status, the energy consumption can be divided into operating energy consumption and standby energy consumption. The operating energy consumption of CNC machine tools and industrial robots includes dynamic energy consumption and fixed energy consumption. Dynamic energy consumption refers to energy consumption that varies significantly with equipment operating parameters, while fixed energy consumption refers to energy consumption that varies less with equipment operating parameters. Taking CNC machine tools as an example, the energy consumption of the spindle and feed drive system is greatly affected by process parameters, which is dynamic energy consumption. The lighting energy consumption and NC system energy consumption are less affected by process parameters and are fixed energy consumption. The operating parameters of quality inspection equipment and conveying equipment are often unchanged, and their operating energy consumption is steady-state energy consumption. The power of equipment during standby is stable and continuous, and its standby energy consumption is only related to standby time, which is also steady-state energy consumption. From the energy footprint composition of workshops, it is necessary to establish the EFMs of workshop equipment in order to establish the workshop EFM. To construct the equipment EFM, its dynamic, fixed, and steady-state energy consumption models need to be established separately.

**Figure 2:**
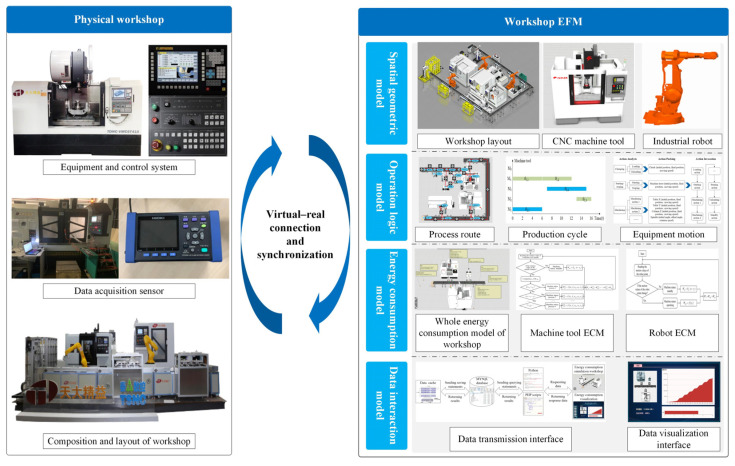
Architecture of EFM for DTW.

The DTW mainly consists of a physical workshop, a virtual workshop, a connection between virtual and real data, and interactive data [24]. The physical workshop is mainly composed of production equipment, the operation control system of the workshop, and the energy consumption during production. The virtual workshop is the mapping of the physical workshop in a digital space, mainly including the spatial geometric model, operational logic model, and ECM of the workshop. By connecting and exchanging data between the virtual and physical workshops, dynamic consistency between the virtual and physical workshops can be achieved. Based on the above analysis, the architecture of an EFM for a DTW is presented, as shown in Figure 2. It mainly consists of the spatial geometric model, operation logic model, ECM, and data interaction model.

### 3.2. Modeling Method of EFM for DTW

This section mainly elaborates on the construction method of an EFM for a DTW. Firstly, the production task of the workshop is analyzed to determine the process route, equipment type, workshop layout, and operation sequence diagram of the equipment. Secondly, based on the equipment operating parameters, the ECM of a single piece of equipment is established. Then, considering the CBMEatWPPL, the whole energy consumption model of the workshop is constructed. Finally, by integrating the spatial geometric model, operational logic model, ECM, and data interaction model of the workshop, the EFM of a DTW is obtained.

The production task analysis roughly involves generating process routes based on product process characteristics, selecting equipment and building the workshop layout, and determining the production cycle and equipment process parameters [25,26]. The spatial geometric model of the workshop mainly includes a geometric model of the workshop layout and equipment. The operation logic model of the workshop mainly consists of the process route, production cycle, and equipment motion. The above models can be constructed using conventional methods [27], which will not be elaborated here.

#### 3.2.1. Construction Method of ECM

(1) Construction of equipment ECM based on multiple linear regression

The energy consumption of a single piece of equipment mainly includes dynamic energy consumption *W_MD_*, fixed energy consumption *W_MR_*, and steady-state energy consumption *W_MS_*, which is formulated as follows:

(1) $$W_{M}=W_{MD}+W_{MR}+W_{MS},$$

where the standby energy consumption is the steady-state energy consumption. Its expression is

(2) $$W_{MS}=P_{MS}\cdot (t_{R}+t_{s}),$$

where *P_MS_* is the standby power, *t_R_* denotes the idle running time, and *t_s_* represents the other standby time. The fixed energy consumption varies less during the equipment operation, and is expressed as follows:

(3) $$W_{MR}=P_{MR}\cdot t_{m},$$

where *P_MR_* denotes the fixed power, and *t_m_* is the equipment operation time.

The precise expression of the dynamic energy consumption of equipment is the key to establishing energy consumption models for equipment and workshops. The dynamic energy consumption varies with changes in the operating parameters and performance status of equipment. The generalized Taylor formula is adopted to establish the dynamic energy consumption model for equipment, which is formulated as follows:

(4) $$W_{MD}=f(m)=a_{0}\cdot m_{1}{\;\;}^{a1}\cdot m_{2}{\;\;}^{a2}\cdot m_{3}{\;\;}^{a3}\cdots ,$$

where *m_i_* represents the equipment operating parameters, and *a_i_* denotes the model coefficients.

A multiple linear regression is applied to obtain the unknown coefficients in the dynamic energy consumption model. Firstly, based on the collected data of operating parameters and energy consumption, the least squares method is used to estimate the unknown coefficients in the dynamic energy consumption model. Then, the collected data are substituted into Equation (4) to calculate the sum of the output errors, determining whether the average error is within the required range. If the requirement is not met, the estimation coefficients need to be adjusted until the average error is less than the maximum allowable average error. The set of coefficients with the smallest average error is regarded as the solution for the unknown coefficients in Equation (4). Finally, the estimated coefficients are substituted into Equation (4) to obtain the multiple linear regression equation of dynamic energy consumption, which is the dynamic energy consumption model.

With the regression analysis, the hypothesis of the linear relationship between the dependent variable and multiple independent variables in the equipment ECM can be tested. The statistical variable *F* is applied to test the significance of regression coefficients, the residual standard deviation σ is adopted to test the output value accuracy of the regression equation, and the average relative error *ε* is used to check if the output value error of the regression equation is within the allowable range of errors [28].

(2) Experimental identification of machine tool ECM considering tool wear

Mainly considering the influences of process parameters and tool wear on the dynamic energy consumption of machine tools, a dynamic energy consumption model of machine tools is established according to Equation (4):

(5) $$W_{mD}=K_{1}\cdot n^{a_{1}}\cdot f^{a_{2}}\cdot a_{p}{\;\;}^{a_{3}}\cdot a_{e}{\;\;}^{a_{4}}+K_{2}\cdot t_{T}\cdot n^{a_{5}}\cdot f^{a_{6}}\cdot a_{p}{\;\;}^{a_{7}}\cdot a_{e}{\;\;}^{a_{8}},$$

where *n*, *f*, *a_p_*, and *a_e_* represent the spindle speed (r/min), feed rate (mm/r), cutting depth (mm), and cutting width, respectively. *t_T_* is the machining time of the tool (min); *K*_1_ and *K*_2_ denote the coefficients related to the machine tool, tool, and workpiece. *a*_1_–*a*_8_ represent exponential parameters of the relationship between the process parameters and dynamic energy consumption.

Orthogonal experiments were conducted to collect machine tool power data. The machine tool was a machining center, the power sensor adopted a clamp-type power meter, the workpiece was made of 45 # steel with dimensions of 60 × 60 × 35 mm, the cutting method was linear milling, and the tool was a four-blade white steel milling cutter with a diameter of 12 mm. The orthogonal experiments were set with four factors and three levels, as shown in Table 1. The machine power under different process parameters and the spindle power and feed drive system power in a stable cutting state of a single milling cutter were collected.

Based on the above experimental data, the dynamic energy consumption for cutting a workpiece with a cutting length of 1 mm on the machine tool was calculated using the multiple linear regression, which is expressed as follows:

(6) $$W_{mD-l}=17.9919\cdot n^{0.0182}\cdot f^{-0.8004}\cdot a_{p}{\;\;}^{0.0791}\cdot a_{e}{\;\;}^{0.1075}+0.0197\cdot t_{T}\cdot n^{0.5586}\cdot f^{-0.1379}\cdot a_{p}{\;\;}^{1.2088}\cdot a_{e}{\;\;}^{1.0918},$$

where

(7) $$t_{T}=\frac{N_{p}\cdot V}{n\cdot f\cdot a_{p}\cdot a_{e}}=\frac{l}{n\cdot f}=\frac{N_{p}\cdot l_{p}}{n\cdot f}.$$

In the above equations, *N_p_* represents the number of processed workpieces by the tool, *V* is the material removal volume from a single workpiece (mm^3^), *l* denotes the total cutting length of workpieces that has been completed by the tool (mm), and *l_p_* is the cutting length of a single workpiece (mm). Then, the dynamic energy consumption model for machining a single workpiece with the machine tool is formulated as follows:

(8) $$\begin{array}{l} W_{mD}=l_{p}\cdot (17.9919\cdot n^{0.0182}\cdot f^{-0.8004}\cdot a_{p}{\;\;}^{0.0791}\cdot a_{e}{\;\;}^{0.1075}\\ +0.0197\cdot N_{p}\cdot l_{p}\cdot n^{-0.4414}\cdot f^{-1.1379}\cdot a_{p}{\;\;}^{1.2088}\cdot a_{e}{\;\;}^{1.0918}) \end{array}.$$

According to Equations (2) and (3), the fixed energy consumption *W_mR_* and standby energy consumption *W_mS_* of the machine tool are formulated as follows:

(9) $$W_{mR}=P_{mR}\cdot t_{m},$$

(10) $$W_{mS}=P_{mS}\cdot (2t_{r}+t_{s}),$$

where *P_mR_* and *P_mS_* are the fixed and standby powers of the machine tool, respectively. Both types of power mainly involve the power of the CNC system, lighting system, and cooling system. *t_m_*, *t_r_*, and *t_s_* denote the operation time, loading time, and other standby time of the machine tool, respectively. As the loading and unloading times of the machine tool are similar, the total loading and unloading time are taken as 2*t_r_*. When the material removal volume from the workpiece is certain, *t_m_* is determined by both the process parameters and material removal volume from the workpiece. Therefore, *t_m_* is expressed as follows:

(11) $$t_{m}=\frac{V}{n\cdot f\cdot a_{p}\cdot a_{e}}$$

Substituting the collected standby power of the machine tool into Equations (9) and (10), respectively, the models of the fixed energy consumption and standby energy consumption of the machine tool are derived as follows:

(12) $$W_{mR}=223.4160\cdot t_{m}=223.4160\cdot \frac{V}{n\cdot f\cdot a_{p}\cdot a_{e}},$$

(13) $$W_{mS}=223.4160\cdot (2t_{r}+t_{s}).$$

Combining Equations (1), (8), (12), and (13), the machine tool ECM is formulated as follows:

(14) $$\begin{array}{l} W_{m}=W_{mD}+W_{mR}+W_{mS}=l_{p}\cdot (17.9919\cdot n^{0.0182}\cdot f^{-0.8004}\cdot a_{p}{\;\;}^{0.0791}\cdot a_{e}{\;\;}^{0.1075}\\ +0.0197\cdot N_{p}\cdot l_{p}\cdot n^{-0.4414}\cdot f^{-1.1379}\cdot a_{p}{\;\;}^{1.2088}\cdot a_{e}{\;\;}^{1.0918})\\ +223.4160\cdot (\frac{V}{n\cdot f\cdot a_{p}\cdot a_{e}}+2t_{r}+t_{s}) \end{array}$$

where the standby time of the machine tool is determined by the operation time of other equipment in the workshop and the workshop’s production cycle.

The regression analysis results of the machine tool ECM are shown in Table 2, where the critical value of the *F*-distribution is obtained by looking up the table [28]. The value of *F* is much greater than its critical value of *F*-distribution, proving that the established machine tool ECM and its parameters are highly significant. By analyzing the residual standard deviation and average relative error, the model error is within the allowable error range. The above analysis results indicate that the machine tool ECM is reliable.

The process route consists of multiple processes, and these processes can be completed by the same tool on the machine tool. According to Equation (8), the dynamic energy consumption of the machine tool to complete the first process is expressed as follows:

(15) $$\begin{array}{l} W_{mD}^{01}=l_{p1}\cdot (17.9919\cdot n_{1}{\;\;}^{0.0182}\cdot f_{1}{\;\;}^{-0.8004}\cdot a_{p1}{\;\;}^{0.0791}\cdot a_{e1}{\;\;}^{0.1075}\\ +0.0197\cdot N_{p1}\cdot l_{p1}\cdot n_{1}{\;\;}^{-0.4414}\cdot f_{1}{\;\;}^{-1.1379}\cdot a_{p1}{\;\;}^{1.2088}\cdot a_{e1}{\;\;}^{1.0918}) \end{array},$$

where *l_p_*_1_ represents the cutting length of the first process, and *N_p_*_1_ is the number of workpieces processed by the tool. Similarly, the dynamic energy consumption of the machine tool in completing the second process is as follows:

(16) $$\begin{array}{l} W_{mD}^{02}=l_{p2}\cdot (17.9919\cdot n_{2}{\;\;}^{0.0182}\cdot f_{2}{\;\;}^{-0.8004}\cdot a_{p2}{\;\;}^{0.0791}\cdot a_{e2}{\;\;}^{0.1075}\\ +0.0197\cdot N_{p2}\cdot l_{p2}\cdot n_{2}{\;\;}^{-0.4414}\cdot f_{2}{\;\;}^{-1.1379}\cdot a_{p2}{\;\;}^{1.2088}\cdot a_{e2}{\;\;}^{1.0918}) \end{array},$$

where *l_p_*_2_ denotes the cutting length of the second process, and *N_p_*_2_ represents the converted number of workpieces processed by the tool in the second process, which can be estimated by the following equation:

(17) $$N_{p2}=N_{p1}+\frac{N_{n2}}{N_{n1}}\cdot N_{p1}-1,$$

where *N_n_*_1_ and *N_n_*_2_ are the number of workpieces that can be processed within the tool life in the first and second processes, respectively.

As the tool wears out during the machining, the energy consumption of a single workpiece in the machine tool gradually increases. Therefore, the dynamic energy consumption generated by the tool during its lifespan is formulated as follows:

(18) $$W_{T}=W_{mD}^{1}+W_{mD}^{2}+W_{mD}^{3}+\ldots +W_{mD}^{N_{n}},$$

where $W_{mD}^{1}$, $W_{mD}^{2}$, and $W_{mD}^{3}$ are the dynamic energy consumption of the first, second, and third workpieces processed by the machine tool, respectively, and *N_n_* is the number of workpieces that the tool can process within its lifespan. Then, the dynamic energy consumption of a single machine tool is expressed as follows:

(19) $$W_{m\_w}=N_{T}\cdot W_{T}+W_{mD}^{1}+W_{mD}^{2}+W_{mD}^{3}+\ldots +W_{mD}^{N_{p}},$$

where *N_T_* is the number of tools consumed by the machine tool.

By combining Equations (12) and (13), the sum of the fixed energy consumption and steady-state energy consumption of the machine tool is formulated as follows:

(20) $$W_{mR}+W_{mS}=223.4160\cdot t,$$

where *t* denotes the total production time in workshop.

By combining Equations (19) and (20), the total energy consumption of the machine tool is derived as follows:

(21) $$W_{m}=W_{m\_w}+W_{mR}+W_{mS}.$$

(3) Experimental identification of robot ECM

**Figure 3:**
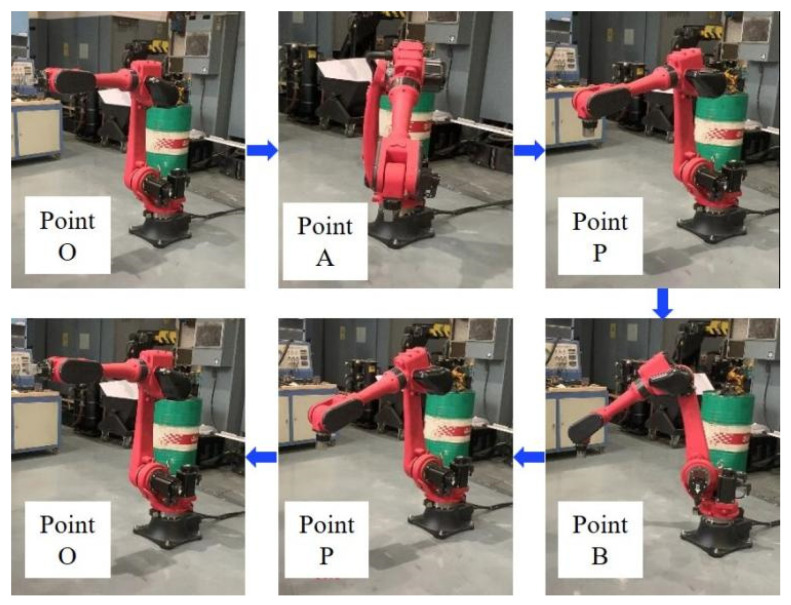
Motion path of robot during loading.

The experiments were carried out to measure robot power. The robot was a six-joint robot, and the power sensor adopted a clamp-type power meter. In order to collect power data of the robot during loading and unloading, the motion trajectory of the robot was planned, as shown in Figure 3. During the loading process, the robot moved from the initial point O to the gripping point A, grabbed the workpiece and moved to the collision-free relay point P in front of the machine tool, then moved to placement point B. After the machine tool completed the clamping of the workpiece, the robot returned to point P and then moved to point O. The unloading path of the robot was consistent with the loading path, with opposite motion directions, and the length of the motion path was the same. Through experimental testing, it was found that the weight change in the workpiece before and after processing had little effect on the robot’s power. Therefore, the power of the robot during the loading process was measured, and the loading and unloading power of the robot were assumed to be symmetrically distributed over time.

Considering the coupling of power between multiple motors of the robot, it was chosen to collect the total power of the robot during motion. The motion mode of the robot was set as a free curve motion. The tool center speed of the robot *ν_T_* was set to increase uniformly from 200 mm/s to 400 mm/s, with each increase being 20 mm/s. The power data of the robot during loading were collected for each group of experiments. The dynamic energy consumption of the robot *W_rW_* for all groups of experiments is shown in Table 3.

According to Table 3, the dynamic energy consumption of the robot is closely related to its tool center speed. Based on Equation (4), a dynamic energy consumption model of the robot is formulated as follows:

(22) $$W_{rW}=K_{3}\cdot v_{T}{\;\;}^{a_{9}},$$

where *K*_3_ denotes the coefficient related to robots and workpieces; *a*_9_ represents the exponential parameter of the relationship between the tool center speed and dynamic energy consumption.

Based on the above experimental data, the dynamic energy consumption of the robot completing one loading process is calculated using the multiple linear regression, which is as follows:

(23) $$W_{rW}=80037.6108\cdot v_{T}{\;\;}^{-0.4682}.$$

According to Equation (2), the standby energy consumption of the robot is derived as follows:

(24) $$W_{rS}=P_{rS}\cdot (t_{m}+t_{s}).$$

Substituting the collected standby power of the robot into Equation (24), the standby energy consumption model of the robot is formulated as follows:

(25) $$W_{rS}=209.5286\cdot (t_{m}+t_{s})=209.5286\cdot (\frac{V}{n\cdot f\cdot a_{p}\cdot a_{e}}+t_{s}).$$

Combining Equations (1), (23), and (25), the robot ECM is derived as follows:

(26) $$W_{r}=2\cdot W_{rW}+W_{rS}=160075.2216\cdot v_{T}{\;\;}^{-0.4682}+209.5286\cdot (\frac{V}{n\cdot f\cdot a_{p}\cdot a_{e}}+t_{s}),$$

where the standby time of the robot is determined by the operation time of other equipment in the workshop and the workshop’s production cycle.

The same method was used to analyze the robot ECM, and the results are shown in Table 4. Similar to the machine tool ECM, it is shown that the robot ECM is correct.

(4) Construction method of whole energy consumption model for workshop

Considering the CBMEatWPPL, the ECMs of each piece of equipment are integrated to obtain the whole energy consumption model for the workshop.

**Figure 4:**
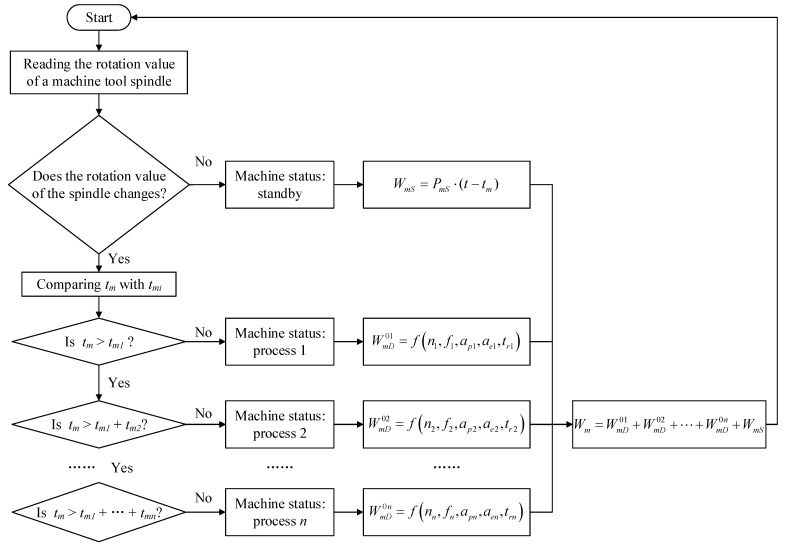
Process for estimating energy consumption of machine tools during workshop operation.

During workshop operations, the status of a machine tool is judged, and the energy consumption of the machine tool is estimated in real time according to Equation (21). As shown in Figure 4, firstly, the rotation value of the machine tool spindle is read. If the value does not change, it is determined that the machine tool is standing by, and the energy consumption of the machine tool is the standby energy consumption; if the value changes, it is judged that the machine tool is operational, and the rotation time of the machine tool spindle is recorded, which is the operation time of the machine tool *t_m_*. Secondly, the cutting time of each process *t_mi_* is calculated and recorded according to Equation (11), which is compared with *t_m_*, to determine which process the machine tool is currently in. The energy consumption of the machine tool is the operating energy consumption of the machine tool in the corresponding process. Finally, the standby energy consumption of the machine tool is added to the operating energy consumption of each process to obtain the total energy consumption of the machine tool.

During workshop operations, the number of times the robot loads and unloads materials is equivalent to the number of processed workpieces. Therefore, the dynamic energy consumption of a robot is formulated as follows:

(27) $$W_{RW}=2\cdot N_{Z}\cdot W_{rW},$$

where *N_Z_* is the number of processed workpieces in the workshop.

According to Equation (25), the standby energy consumption of the robot is expressed as follows:

(28) $$W_{rS}=209.5286\cdot (t-2\cdot N_{Z}\cdot \frac{L}{v_{T}}),$$

where *L* represents the tool center displacement of the robot.

By summing Equations (27) and (28), the total energy consumption of the robot is derived as follows:

(29) $$W_{r}=W_{RW}+W_{rS}.$$

**Figure 5:**
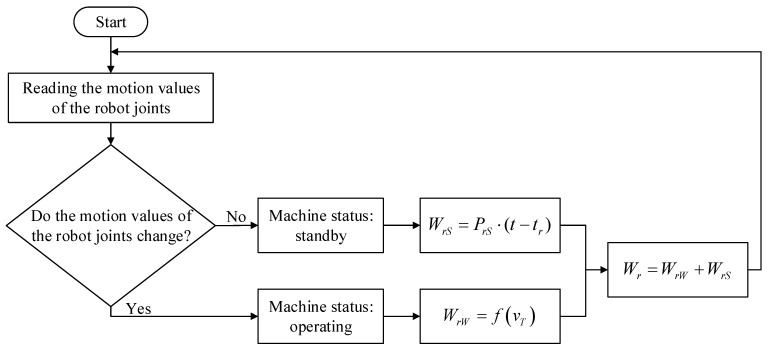
Process for estimating energy consumption of robots during workshop operation.

The real-time estimation process of robot energy consumption is similar to that of machine tool energy consumption, as shown in Figure 5. The motion values of the six joints of the robot are read. If the motion values of all joints have not changed, it is determined that the robot is standing by, and the robot energy consumption is the standby energy consumption; if the motion value of any joint of the robot changes, it is judged that the robot is in operation, and the robot energy consumption is the operating energy consumption. The standby energy consumption of the robot is summed up with the operating energy consumption to obtain the total energy consumption of the robot during workshop operations.

The total energy consumption of quality inspection equipment during workshop operations is formulated as follows:

(30) $$W_{b}=W_{bW}+W_{bS}=N_{Z}\cdot t_{b}\cdot P_{b}{\;\;}_{W}+(t-N_{Z}\cdot t_{b})\cdot P_{b}{\;\;}_{S},$$

where *W_bW_* and *W_bS_* are the operating energy consumption and standby energy consumption of the quality inspection equipment, respectively. *t_b_*, *P_bW_*, and *P_bS_* denote the operation time, operating power, and standby power of the quality inspection equipment, respectively. The quality inspection equipment belongs to non-processing equipment, and its operating energy consumption and standby energy consumption can be regarded as steady-state energy consumption. Therefore, the energy consumption of the quality inspection equipment is calculated with its operating power, standby power, and operation time.

The conveying equipment operates continuously during workshop operations, and its energy consumption is expressed as follows:

(31) $$W_{c}=t\cdot P_{c},$$

where *P_c_* is the operating power of the conveying equipment. The conveying equipment is also non-processing equipment, and its operating energy consumption can be regarded as steady-state energy consumption. Therefore, its energy consumption is calculated with the operating power and operation time.

The process of real-time energy consumption estimation for the quality inspection equipment and conveying equipment is similar to that of machine tools and robots, and will not be repeated here.

Based on the above equations, the whole energy consumption model of the workshop is derived as follows:

(32) $$W_{W}=\sum_{i=1}^{x_{m}}W_{mi}+\sum_{i=1}^{x_{r}}W_{ri}+\sum_{i=1}^{x_{b}}W_{bi}+\sum_{i=1}^{x_{c}}W_{ci}+W_{o},$$

where *W_mi_*, *W_ri_*, *W_bi_*, and *W_ci_* represent the energy consumption of the *i*-th machine tool, robot, quality inspection equipment, and conveying equipment, respectively. *W_o_* is the other fixed energy consumption in the workshop. *x_m_*, *x_r_*, *x_b_*, and *x_c_* denote the number of machine tools, robots, pieces of quality inspection equipment, and pieces of conveying equipment in the workshop, respectively.

With the support of the DTW, this ECM will adjust the estimation method of workshop energy consumption in real time according to the changes in workshop operation and equipment status, ensuring dynamic consistency between the estimated and actual energy consumption of the workshop.

#### 3.2.2. Construction Method of Data Interaction Model

The dynamic consistency between the EFM and physical workshop is primarily achieved through data transmission and interaction in the DTW. This section mainly elaborates on the construction of the data interaction model.

(1) Hierarchical structure of data interaction model

**Figure 6:**
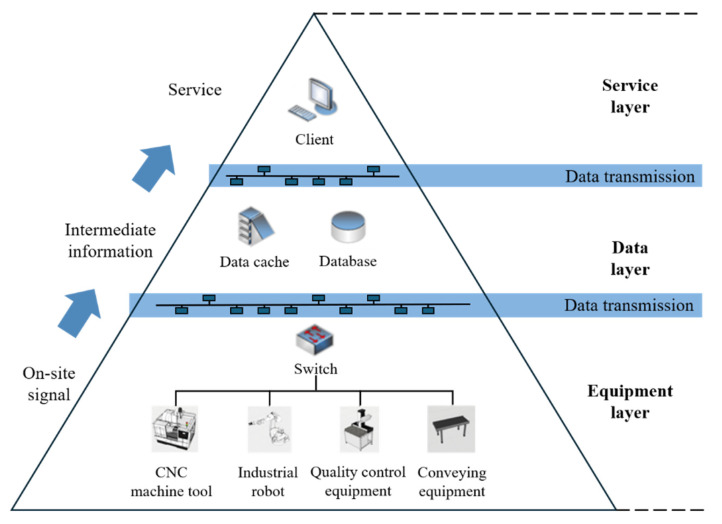
Hierarchical structure of data interaction model.

As shown in Figure 6, the hierarchical structure of the data interaction model in the DTW is mainly composed of three layers: the equipment layer, data layer, and service layer. The equipment layer includes equipment that generates data, sensors that collect data, and switches that transmit data. The data layer is primarily composed of a data cache for receiving data and a database for storing data. The service layer mainly consists of data analysis and visualization. These three layers are connected and interact with each other through data transmission.

The workshop consists of various pieces of equipment, such as machine tools and robots. There are multisource data, such as sensor data and CNC system data, that need to be collected. Therefore, a universal data transmission form is developed in the equipment layer to collect the multisource data in the workshop. In order to obtain the workshop data and transmit effective information to the service layer, data acquisition, processing, and storage are completed in the data layer. In the service layer, the data analysis and visualization are realized, and a unified approach is used to access and query data in the database.

(2) Data collection and transmission

Based on the above hierarchical structure, the data collection and transmission are developed. At the equipment layer, industrial switches are used to connect various pieces of equipment and sensors in the workshop, forming a workshop LAN. Considering the real time of data collection, a data transmission interface between the workshop LAN and the data layer is set up. The collected data are transmitted to the data cache through the TCP/IP protocol.

**Figure 7:**
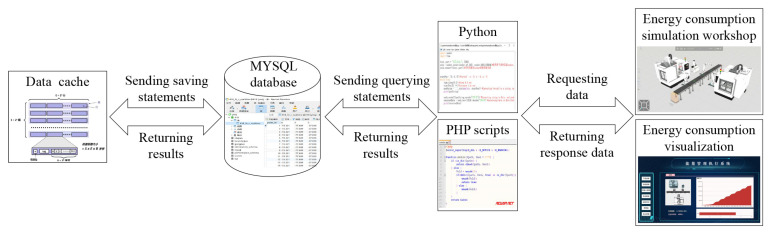
Data transmission interface of the data layer.

In the data layer, an interface between the data cache and the database is built, as shown in Figure 7. The data cache is developed using Visual Studio 2019, and the database adopts MySQL 8.0. Scripts of C++ 6.0 are programmed to connect the data cache to the MySQL database, and SQL statements are written to complete the data addition, deletion, and modification in the database. The MySQL database is connected to the simulation workshop of energy consumption through scripts of Python 3.9. SQL statements are written to query and write real-time data in the database.

At the service layer, an interface between the service layer and MySQL database is developed through PHP scripts. A data query request is sent to the database. If the corresponding data are found, the associated array of the query results is obtained for data analysis and visualization, and the result data in JSON format are returned. If no corresponding data are found, “No data found” is returned.

(3) Visualization of energy consumption data

Based on the workshop ECM, the real-time estimation of workshop energy consumption is completed through the real-time collection of workshop data. Firstly, based on the workshop’s process route, the operation process of the equipment is segmented, and the equipment energy consumption during each stage is estimated separately. Then, according to the CBMEatWPPL, the whole energy consumption of the workshop is calculated. Finally, the energy consumption of the workshop and equipment are visualized.

According to the ECM in Section 3.2.1, an estimation function for machine tool energy consumption during workshop operations is formulated as follows:

(33) Wm(t)=PmR⋅t0<t<t1  PmR⋅t+f(n1,f1,ap1,ae1,tT1)⋅n1⋅f1⋅(t−t1)lp1t1<t<t1+lp1n1⋅f1  PmR⋅t+f(n1,f1,ap1,ae1,tT1) +f(n2,f2,ap2,ae2,tT2)t1+lp1n1⋅f1<t⋅n2⋅f2⋅(t−t1−lp1n⋅f)lp2<t1+lp1n1⋅f1+lp2n2⋅f2  ……,

where *t*_1_ is the time when the machine tool starts running.

Similarly, an energy consumption estimation function for robots is expressed as follows:

(34) $$W_{r}(t)=\left\{\begin{array}{ll} P_{rS}\cdot t & 0<t<t_{2}\\ P_{rS}\cdot t+f(v_{T})\cdot \frac{v_{T}\cdot (t-t_{2})}{L} & t_{2}<t<t_{2}+\frac{L}{v_{T}}\\ \ldots & \ldots \end{array}\right.,$$

where *t*_2_ denotes the time when the robot starts running.

The energy consumption estimation functions of various pieces of equipment in the workshop are summed up in the time dimension to obtain the estimation function of the whole energy consumption in the workshop. This function can be used to characterize the dynamic changes in workshop energy consumption. The real-time energy consumption data can be written into the database with the data collection and transmission.

The data visualization is achieved by combining JavaScript scripts and Ajax technology. Firstly, the JSON string of the data in the PHP script is obtained and converted into the corresponding JavaScript object. Secondly, the algebraic operations on the obtained data are performed, and the results are stored in an array in chronological order. A polling method is adopted to read database data to ensure the real time of data queries. Finally, the energy consumption data are visualized through Echart.

Given the data interaction model, the transmission of virtual and real data in the DTW can be achieved, as well as the internal data exchange in the workshop EFM.

## 4. Case Study

Based on the production of a certain workpiece, a production unit that consists of various machine tools and robots was built. A workshop EFM for this production unit was constructed. On this basis, the cross-equipment process parameters of the production unit were collaboratively optimized to reduce its energy consumption. The feasibility of the proposed method was validated.

### 4.1. Case Description and Analysis

**Figure 8:**
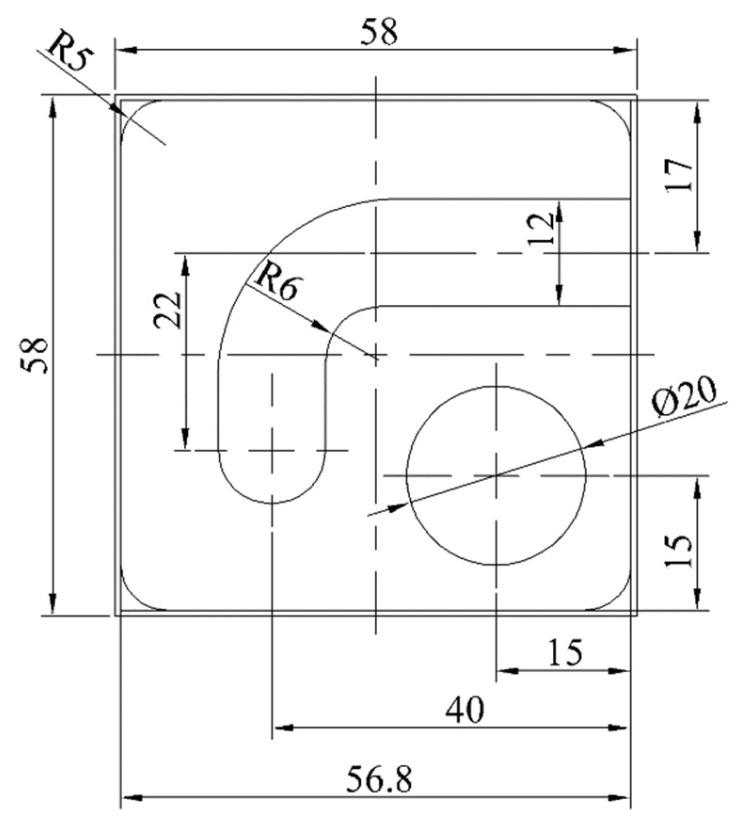
Processed workpiece.

**Figure 9:**
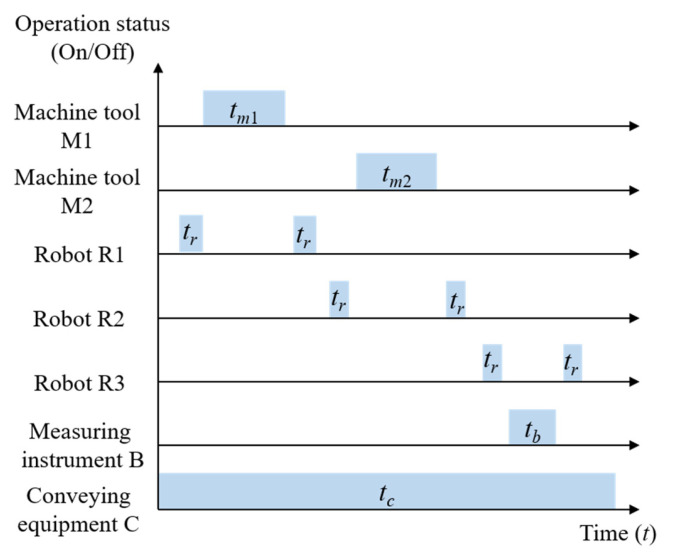
Equipment operating sequences.

As shown in Figure 8, the process features of the workpiece primarily include a surface, groove, hole, and rounded corner. The selected processing method is rough machining, and a CNC milling machine is selected as the processing equipment. Taking into account the process route and existing production resources, two CNC milling machines of the same type are selected to configure the production unit in parallel. Three industrial robots are adopted to load and unload workpieces, and a coordinate measuring instrument is applied to measure the workpiece dimensions. Through experiments, it was found that when the first machine tool completed milling the surface and hole, the second machine tool completed milling the rounded corner and groove. According to this, the equipment operating sequences in the production unit were determined, as shown in Figure 9.

### 4.2. EFM of Production Unit

**Figure 10:**
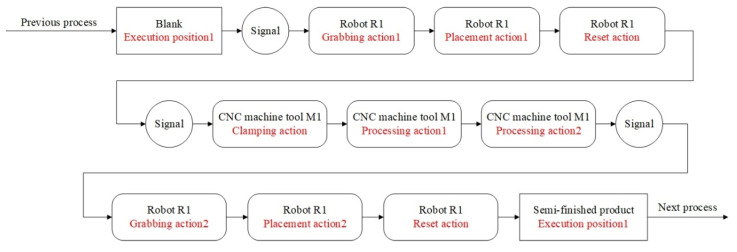
Operation logic model.

The EFM of the production unit was constructed using the method in Section 3.2. Based on the process route and equipment operating sequence, the operation logic model of the production unit was established. Taking a production unit composed of machine tool M1 and robot R1 as an example, its operation logic model is shown in Figure 10. Similarly, by adding the operating sequence of other equipment in the production unit, the operation logic model of the production unit could be obtained. The spatial geometric model and operation logic model of the production unit were integrated in a commercial software to enable the virtual action simulation.

Using the method in Section 3.2.1, the whole energy consumption model for the production unit was formulated as follows:

(35) $$W=W_{m}^{M1}+W_{m}^{M2}+3W_{r}^{R}+W_{b}^{B}+W_{c}^{C}+W_{o},$$

where $W_{m}^{M1}$, $W_{m}^{M2}$, and $W_{r}^{R}$ represent the energy consumption of machine tool M1, machine tool M2, and the single robot, respectively. $W_{b}^{B}$ and $W_{c}^{C}$ denote the energy consumption of the coordinate measuring instrument and conveying equipment, respectively. *W_o_* is the other fixed energy consumption.

**Figure 11:**
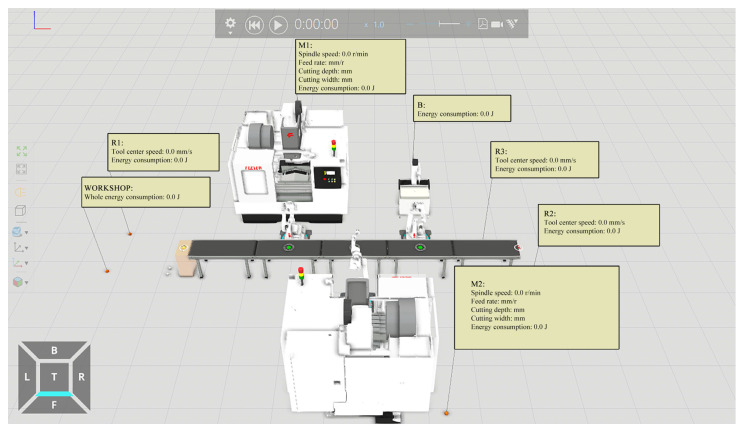
Energy consumption simulation model of the production unit.

**Figure 12:**
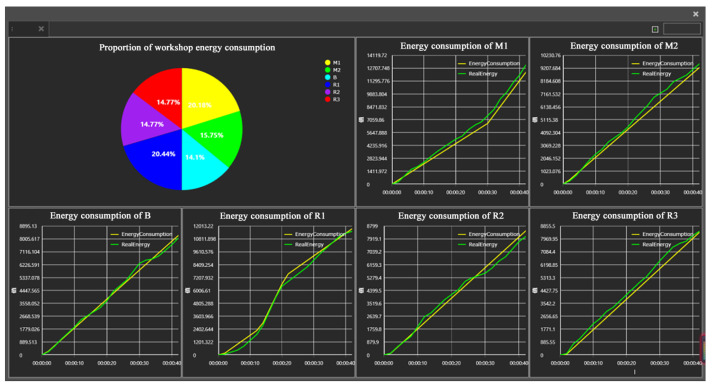
Statistical analysis of equipment energy consumption.

The spatial geometric model, operational logic model, and energy consumption model of the production unit were integrated into the commercial software to obtain the energy consumption simulation model of the production unit, as shown in Figure 11. Taking the production process of a single workpiece as an example, this model could simulate and calculate the energy consumption of each piece of equipment in the production unit as well as the whole energy consumption. A statistical analysis on the energy consumption of the equipment and the production unit could be conducted. As shown in Figure 12, the pie chart shows the proportion of the energy consumption of each piece of equipment in the whole energy consumption of the production unit. The energy consumption curves of each piece of equipment specifically include simulated and real energy consumption curves. It can be seen that the change trend in the energy consumption obtained by the simulation model is basically consistent with that in the real energy consumption.

Using the method in Section 3.2.2, the data interaction model for the production unit was established, which was then integrated with the energy consumption simulation model to obtain the EFM of the production unit. Driven by the real-time data, the EFM can characterize the fluctuations in equipment energy consumption and dynamic changes in the whole energy consumption in the production unit. All in all, the EFM can maintain dynamic consistency with the production unit entity in terms of equipment status and motion, operation logic, and energy consumption.

**Figure 13:**
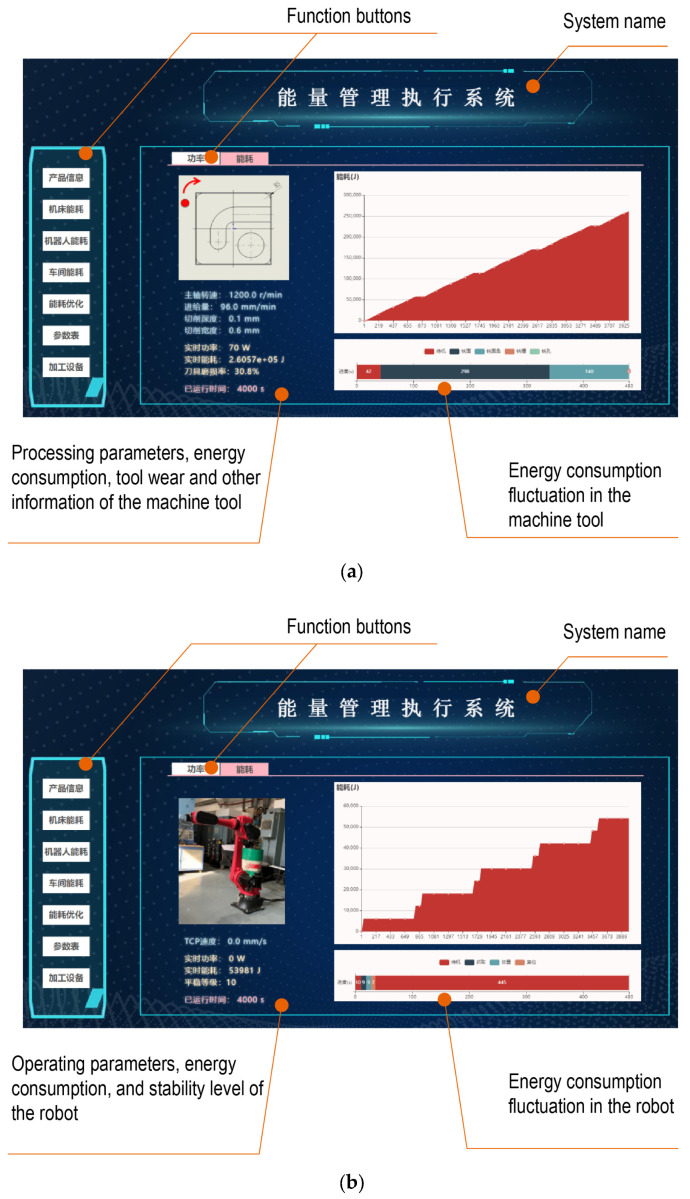

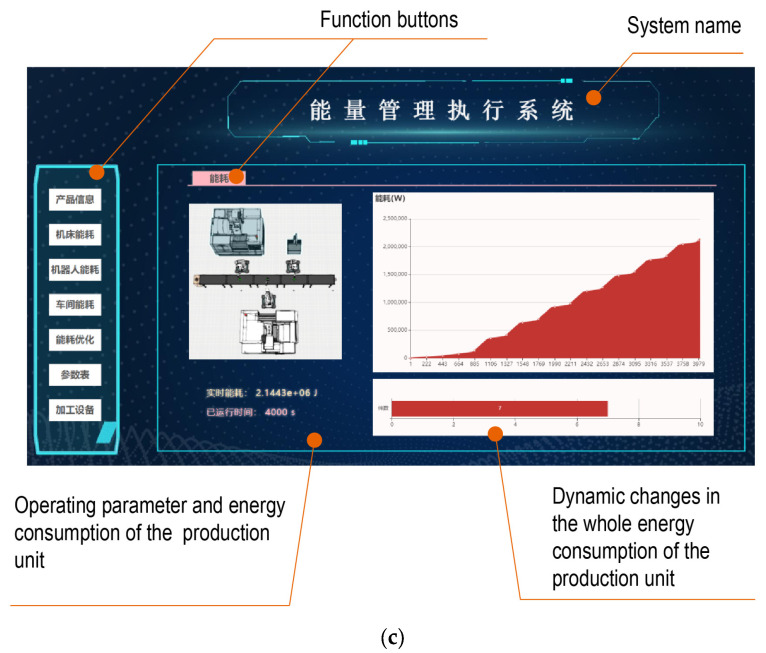
Visualization interface for production unit EFM. (**a**) Energy consumption of machine tool. (**b**) Energy consumption of robot. (**c**) Energy consumption of production unit.

Through HTML5, a front-end interface was self-developed to visualize the dynamic changes in the energy consumption, operation status, and production progress of the production unit. The energy consumption of the equipment and the production unit obtained through the EFM are shown in Figure 13. The energy consumption fluctuations in the machine tool and robot are intuitively displayed in Figure 13a and Figure 13b, respectively. The dynamic changes in the whole energy consumption of the production unit are shown in Figure 13c. In addition, the real-time information on the machining process and tool wear of machine tools, the tool center speed and motion stability of the robot, the production progress of the production unit, and the number of completed workpieces is also displayed.

### 4.3. Collaborative Optimization of Cross-Equipment Process Parameters Based on EFM

The workshop EFM reflects the dynamic mapping relationship between the equipment process parameters and workshop energy consumption. Therefore, based on the EFM, the process parameters of the workshop equipment were optimized to reduce workshop energy consumption, thereby proving the superiority of the EFM.

The process route in a workshop usually includes various processes, involving multiple pieces of equipment, and requires the collaboration of process parameters of multiple pieces of equipment during the workshop’s production process. In addition, the power and duration of different types of equipment are different, indicating that different process parameters have different impacts on the workshop energy consumption. Therefore, it is necessary to analyze the energy consumed by each piece of equipment completing each process and select the process with the highest proportion of energy consumption as the main optimization sequence.

In order to comprehensively improve various performances in the workshop production process, other key factors that affect the production efficiency and product quality should also be considered. According to engineering experience, excessive tool wear can lead to a decrease in product quality and an increase in tool consumption. Unstable robot movement can cause an increase in defective products. Excessive processing time of workpieces can result in low production efficiency. Therefore, taking tool life, robot motion stability, production time, and workshop energy consumption as optimization objectives, a multi-objective optimization function for the workshop was formulated and solved to obtain the optimal process parameters of various pieces of equipment in the workshop.

#### 4.3.1. Objective 1: Workshop Energy Consumption

As mentioned above, the dynamic mapping relationship between equipment process parameters and workshop energy consumption is mainly reflected by the dynamic energy consumption in the EFM. Therefore, dynamic energy consumption in workshop was taken as the energy consumption objective, primarily including the dynamic energy consumption of machine tools and robots.

Usually, the specific energy consumption is used to express the energy utilization rate during processing. For machine tools, the specific energy consumption refers to the ratio of the cutting process energy consumption to the volume of removed material [29]. According to Equation (5), the dynamic energy consumption of the machine tool is the energy consumption of cutting a fixed length of the workpiece. Therefore, by dividing the dynamic energy consumption of the machine tool by the area of the cutting surface (cutting depth multiplied by cutting width), one can obtain the dynamic specific energy consumption of the tool for removing the same material volume. The dynamic specific energy consumption model of the machine tool is expressed as follows:

(36) $${W^{\prime }}_{mD}=K_{1}\cdot n^{a_{1}}\cdot f^{a_{2}}\cdot a_{p}{\;\;}^{a_{3}-1}\cdot a_{e}{\;\;}^{a_{4}-1}+K_{2}\cdot t_{T}\cdot n^{a_{5}}\cdot f^{a_{6}}\cdot a_{p}{\;\;}^{a_{7}-1}\cdot a_{e}{\;\;}^{a_{8}-1}.$$

Substituting the dynamic energy consumption of the machine tool into Equation (36), the dynamic specific energy consumption of the machine tool is formulated as follows:

(37) $${W^{\prime }}_{mD}=l_{p}\cdot \left(\begin{array}{l} 17.9919\cdot n^{0.0182}\cdot f^{-0.8004}\cdot a_{p}{\;\;}^{-0.9209}\cdot a_{e}{\;\;}^{-0.8925}+\\ 0.0197\cdot N_{p}\cdot l_{p}\cdot n^{-0.4414}\cdot f^{-1.1379}\cdot a_{p}{\;\;}^{0.2088}\cdot a_{e}{\;\;}^{0.0918} \end{array}\right).$$

For robots, the specific energy consumption refers to the ratio of the operation process energy consumption to the tool center displacement. According to Equation (23), the dynamic energy consumption of the robot is the energy consumption required by the robot to complete one loading process. Since the tool center displacements for each loading process are the same, the dynamic energy consumption of the robot is equivalent to its dynamic specific energy consumption. Therefore, the dynamic specific energy consumption of the robot is expressed as follows:

(38) $${W^{\prime }}_{rW}=80037.6108\cdot v_{T}{\;\;}^{-0.4682}.$$

By adding up the dynamic specific energy consumption of machine tools and robots, the dynamic specific energy consumption of the workshop is derived as follows:

(39) $$W_{D}=\sum_{i=1}^{x_{m}}{W^{\prime }}_{mDi}+2\sum_{j=1}^{x_{r}}{W^{\prime }}_{rWj},$$

where ${W^{\prime }}_{mDi}$ and ${W^{\prime }}_{rWj}$ represent the dynamic specific energy consumption of the *i*-th machine tool and *j*-th robot in the workshop, respectively. *x_m_* and *x_r_* are the number of machine tools and robots in the workshop, respectively. The dynamic specific energy consumption of the workshop is taken as objective 1.

#### 4.3.2. Objective 2: Tool Life

The tool life is related to the degree of tool wear. The ways to characterize the degree of tool wear mainly include the rake face wear, flank wear, comprehensive consideration of the front and rear tool faces, and tool edge passivation. The flank wear was selected to characterize the tool life. The total cutting length that a tool can complete within its lifespan is a function related to the process parameters, which is expressed as follows:

(40) $$l_{n}=f(n,f,a_{p},a_{e}).$$

**Figure 14:**
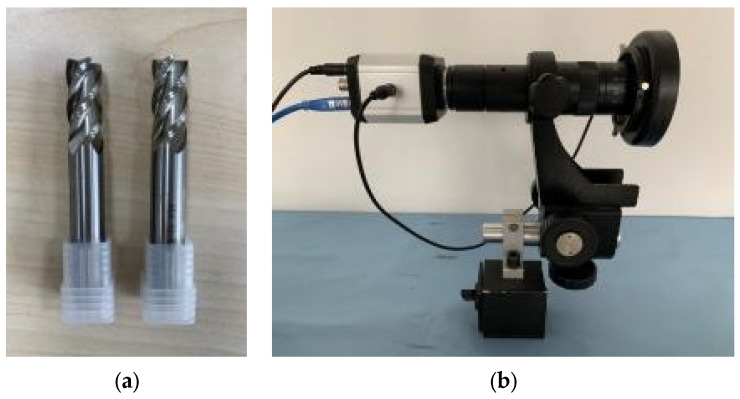
(**a**) White steel milling cutter and (**b**) microscope.

**Figure 15:**
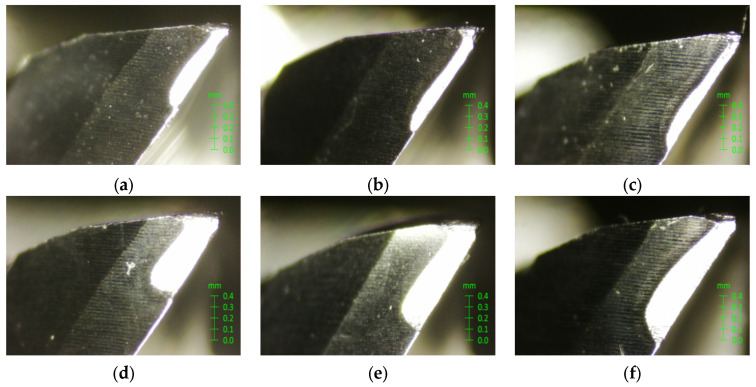
Microscopic images of flank wear. (**a**) Tool 1 cutting 3780 mm; (**b**) Tool 2 cutting 3360 mm; (**c**) Tool 3 cutting 2880 mm; (**d**) Tool 1 cutting 8820 mm; (**e**) Tool 2 cutting 5880 mm; (**f**) Tool 3 cutting 5400 mm.

In the orthogonal experiments shown in Table 1, the tool life under various cutting conditions was measured. The tool parameters and microscope type are shown in Table 5. The white steel milling cutter and microscope are shown in Figure 14. The microscope images of the flank wear measured in the experiments are shown in Figure 15.

Based on the generalized Taylor formula, the relationship between the total cutting length of the tool within its lifespan and process parameters is formulated as follows:

(41) $$l_{n}=K_{4}\cdot n^{a_{10}}\cdot f^{a11}\cdot a_{p}{\;\;}^{a12}\cdot a_{e}{\;\;}^{a13},$$

where *K*_4_ is the coefficient related to the tool and workpiece materials; *a*_10_–*a*_13_ denote the exponential parameters of the relationship between various process parameters and the tool life. Based on the orthogonal experimental data, the tool life model was formulated through multiple linear regression, which is as follows:

(42) $$l_{n}=5995.6248\cdot n^{-0.2265}\cdot f^{-0.6254}\cdot a_{p}{\;\;}^{0.6961}\cdot a_{e}{\;\;}^{0.6506},$$

where the unit of *l_n_* is mm.

The number of workpieces that the tool can process within its lifespan is expressed as follows:

(43) $$N_{n}=\frac{5995.6248}{l_{p}}\cdot n^{-0.2265}\cdot f^{-0.6254}\cdot a_{p}{\;\;}^{0.6961}\cdot a_{e}{\;\;}^{0.6506},$$

which is set as objective 2.

#### 4.3.3. Objective 3: Robot Motion Stability

According to engineering experience, sudden changes in the direction of the tool center motion of the robot during motion, or significant speed changes during the start and stop of the robot, can cause significant acceleration at the tool center, leading to unstable clamping of the workpieces. Therefore, if the acceleration of the robot tool center is low during motion, it is considered that the robot’s motion stability is high.

The acceleration of the robot tool center is determined by the torque of various servo motors. The maximum torque of the servo motors during operation is related to the maximum power. Therefore, the maximum total power of the robot during motion is considered the evaluation index for the robot’s motion stability.

Based on the generalized Taylor formula, the relationship between the maximum power of the robot and the tool center speed was formulated as follows:

(44) $$P_{\max}=K_{5}\cdot v_{T}{\;\;}^{a_{14}},$$

where *K*_5_ is the coefficient related to the robots and workpieces; *a*_14_ denotes the exponential parameter of the relationship between the maximum power and the tool center speed.

The maximum power of the robot completing a loading process at different tool center speeds was measured, and the results are shown in Table 6. Based on the experimental data, the maximum power model of the robot was formulated using multiple linear regression, which is as follows:

(45) $$P_{\max}=26.7912\cdot v_{T}{\;\;}^{0.4852}.$$

The maximum power model of the robot completing one loading process was considered objective 3.

#### 4.3.4. Objective 4: Production Time

The average production time of a single workpiece is related to the equipment process parameters, as well as the amount of equipment participating in production simultaneously. Since the process parameter was set as the optimization variable, it was necessary to exclude the impact of the equipment amount on the production time.

In a production unit consisting of a machine tool and a robot, the production time of a single workpiece is expressed as follows:

(46) $$t_{p}=t_{m}+2t_{r}+t_{s}.$$

Substituting Equation (11) and the robot operation time into Equation (46), the production time model of a single workpiece is derived as follows:

(47) $$t_{p}=\frac{V}{n\cdot f\cdot a_{p}\cdot a_{e}}\cdot 60+2\cdot \frac{L}{v_{T}}+t_{s}=\frac{l_{p}}{n\cdot f}\cdot 60+2\cdot \frac{L}{v_{T}}+t_{s}.$$

where *t_D_* represents the dynamic production time varying with process parameters, which is expressed as follows:

(48) $$t_{D}=\frac{l_{p}}{n\cdot f}\cdot 60+2\cdot \frac{L}{v_{T}}.$$

where *t_D_* is considered objective 4.

#### 4.3.5. Discussion

Based on the four optimization objectives of workshop energy consumption, tool life, robot motion stability, and production time, a multi-objective function for the collaborative optimization of cross-equipment process parameters was formulated using the linear weighted sum method. When the workshop energy consumption, maximum robot power, and production time are low, they meet the optimization expectations. When the tool life is high, it meets the optimization expectation. Therefore, the workshop energy consumption, robot motion stability, and production time were normalized, and the tool life was normalized after taking the reciprocal. The multi-objective optimization function is formulated as follows:

(49) $$F(n,f,a_{p},a_{e},v_{T})=\omega_{1}\cdot {W_{D}}^{*}+\omega_{2}\cdot {\left({N_{n}}^{-1}\right)}^{*}+\omega_{3}\cdot P_{\max}{\;\;}^{*}+\omega_{4}\cdot {t_{D}}^{*},$$

where W_D_^*^, *P*_max_^*^, t_D_^*^, and (*N_n_*^−1^)^*^ represent the normalized workshop energy consumption, robot motion stability, production time, and tool life, respectively. *ω*_1_, *ω*_2_, *ω*_3_, and *ω*_4_ denote the weights of these four optimization objectives. The weight values were determined based on different optimization focuses.

The main optimization objective here is the energy consumption of the production unit; therefore, a larger weight of objective 1 is set. The weight of each objective is shown in Table 7, and the ranges of equipment process parameters are shown in Table 8.

**Figure 16:**
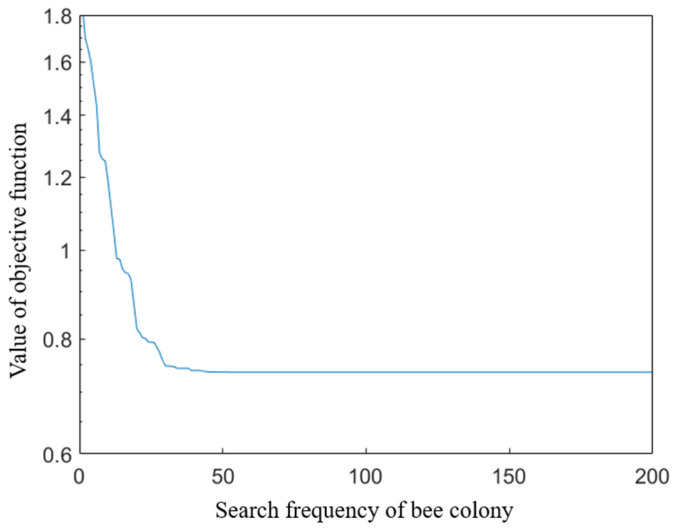
Solving process of bee colony algorithm for multi-objective optimization function.

The bee colony algorithm is adopted to solve the multi-objective optimization function. A total of 100 bees are selected, including 20 leading bees and 80 accompanying bees. A reconnaissance module is set up to prevent the optimization results from falling into local optima. The search frequency of the bee colony is 200 times, and a total of five search rounds are conducted. When the *F* in Equation (49) reaches the optimization expectation, the parameters are the optimization results of cross-equipment process parameters. The solution process is shown in Figure 16. The optimization results of the process parameters are shown in Table 9, where the process parameters before optimization are commonly used values in engineering.

The workpiece shown in Figure 8 is processed using optimized equipment process parameters. A comparison of the results before and after optimization is shown in Table 10. *n_R_* is the reference value for evaluating the optimization results, which is expressed as follows:

(50) $$n_{R}=\frac{\left|R_{c}-I\right|-R_{l}}{R_{u}-R_{l}}$$

where *R_c_* is the theoretical indicator value, *I* represents the value of the optimization objective, and *R_u_* and *R_l_* denote the upper and lower limits of the indicator interval, respectively.

From Table 10, the dynamic energy consumption of machine tools M1 and M2 is reduced after optimization. The optimization effect of machine tool M1 for milling surfaces and holes is more obvious. The reason is that the optimized process parameters shorten the processing time, leading to a significant reduction in energy consumption. The process parameters of machine tool M2 for milling grooves and rounded corners do not change much before and after optimization, so the energy consumption is less reduced. After optimization, the operating energy consumption of robots and the production time are also reduced. The production time decreased by 22.77% compared to that before optimization. Due to the increase in the feed rate and cutting width of the machine tool, the number of cutting workpieces within the tool life is reduced. The increase in the tool center speed of the robot resulted in an increase in the robot maximum power.

**Figure 17:**
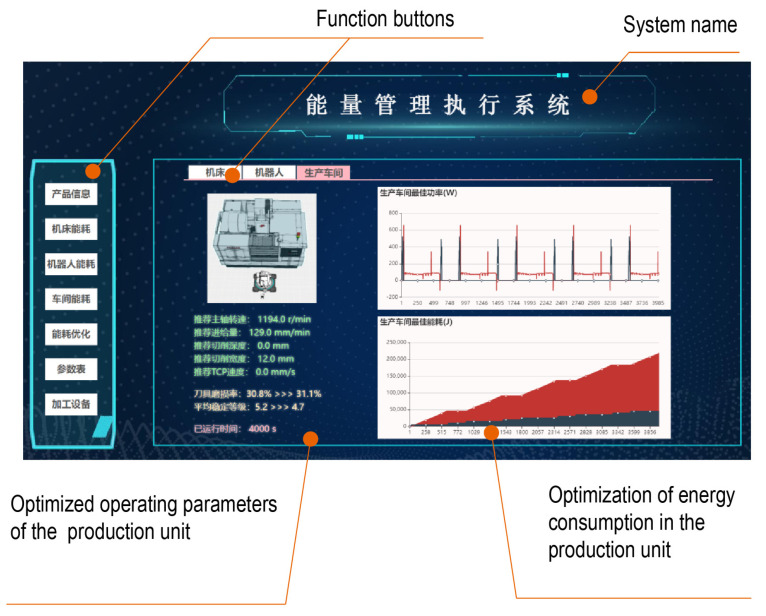
Energy consumption optimization interface for the production unit.

According to the production unit EFM, the whole energy consumption of the production unit before and after optimization is 1590.5 kJ and 1239.1 kJ, respectively. The energy consumption savings rate is 22.09%. The optimization of energy consumption in the production unit can also be visualized through the front-end interface, as shown in Figure 17. The figure shows the optimized values of the equipment process parameters and depicts the whole energy consumption of the production unit before and after optimization.

## 5. Conclusions

Reducing workshop energy consumption is one of the important means for manufacturing enterprises to save energy and reduce emissions. In order to address the difficulty of accurately characterizing the fluctuations in equipment energy consumption and the dynamic evolution of whole energy consumption in low-carbon workshops, a low-carbon operation-oriented construction method of an EFM for a DTW is proposed in this paper. By comprehensively considering the fluctuations in equipment energy consumption and the CBMEatWPPL, an EFM for a production unit is established and verified through a case study. A summary of the major findings of this study is as follows:

(1) The construction method of an EFM for a DTW is proposed. By analyzing the energy composition of the DTW, the definition and architecture of the EFM for the DTW are presented. With a focus on considering the impact of fluctuations in equipment energy consumption, an ECM for a single piece of equipment is established. On this basis, considering the CBMEatWPPL, a whole energy consumption model for a workshop is constructed. By integrating the spatial geometric model, operational logic model, ECM, and data interaction model of the workshop, the EFM of the DTW is obtained. Taking a production unit as a case, its EFM is constructed with the proposed method. The characterization and visualization of the fluctuations in the equipment energy consumption and dynamic changes in the whole energy consumption of the product unit are realized.
(2) The EFM-based collaborative optimization of cross-equipment process parameters is completed. Taking the production unit as the case, an objective function of the workshop energy consumption is formulated according to the EFM. With workshop energy consumption as the main objective and tool life, robot motion stability, and production time as secondary objectives, the cross-equipment process parameters are collaboratively optimized using the bee colony algorithm. By comparing the experimental results before and after optimization, it was found that the energy consumption of a single machine tool was reduced, the number of processed workpieces within the tool life was reduced, the production time was reduced by 22.77%, and the whole energy consumption of the production unit was reduced by 22.09%. In summary, the optimized process parameters achieved a significant reduction in production time and whole workshop energy consumption while losing a small amount of tool life. This once again proves the superiority of the EFM.

Future research will focus on integrating the production process, scheduling plan, and equipment process parameter to further improve the optimization of workshop energy consumption.

## Figures and Tables

**Table 1:** Parameters of the orthogonal experiments.

No.	*n* (r/min)	*f* (mm/r)	*a_p_* (mm)	*a_e_* (mm)
1	1000	0.10	0.8	0.4
2	1000	0.11	1.0	0.5
3	1000	0.12	1.2	0.6
4	1100	0.10	1.0	0.6
5	1100	0.11	1.2	0.4
6	1100	0.12	0.8	0.5
7	1200	0.10	1.2	0.5
8	1200	0.11	0.8	0.6
9	1200	0.12	1.0	0.4

**Table 2:** Regression analysis of machine tool ECM.

Evaluating Indicator	Indicator Values	
	Regression	Residual error
Square sum	1.4078 × 10^8^	7.3816 × 10^6^
Freedom	5	163
Mean square	2.8156 × 10^7^	4.5286 × 10^4^
*F*	621.74	
Critical value of *F*-distribution (Confidence interval *α* = 0.01)	3.02	
*σ*	212.81	
*ε*	2.01%	

**Table 3:** Experimental data on dynamic energy consumption of robot.

No.	*ν_T_* (mm/s)	*W_rW_* (J)
1	200	6588.9
2	220	6405.7
3	240	6177.2
4	260	5971.8
5	280	5665.8
6	300	5622.3
7	320	5448.2
8	340	5278.7
9	360	5086.6
10	380	4776.1
11	400	4563.6

**Table 4:** Regression analysis of robot ECM.

Evaluating Indicator	Indicator Values	
	Regression	Residual error
Square sum	3.7219 × 10^6^	1.4417 × 10^5^
Freedom	1	9
Mean square	3.7219 × 10^6^	1.6019 × 10^4^
*F*	232.34	
Critical value of *F*-distribution (Confidence interval *α* = 0.01)	10.04	
*σ*	126.57	
*ε*	1.48%	

**Table 5:** Tool parameters and acquisition equipment type in orthogonal experiment.

Equipment	Equipment Information	
White steel milling cutter	Material quality	M2AL aluminum containing high-speed steel
	Number of cutting edges	4
	Diameter	12 mm
Microscope	Type	XDS-10A

**Table 6:** Collected data of robot maximum power.

No.	*ν_T_* (mm/s)	*P*_max_ (W)
1	200	350.3
2	220	367.2
3	240	382.7
4	260	398.4
5	280	395.4
6	300	402.8
7	320	406.0
8	340	472.9
9	360	482.4
10	380	483.6
11	400	486.8

**Table 7:** Weight of optimization objectives.

No.	*ω*_1_	*ω*_2_	*ω*_3_	*ω*_4_
Weight value	2.0	6.0	0.1	1.0

**Table 8:** Range of equipment process parameters.

Process Parameter	Range	Process Parameter	Range
*n*_1_ (r/min)	1000–1200	*n*_3_ (r/min)	800–960
*f*_1_ (mm/r)	0.100–0.120	*f*_3_ (mm/r)	0.080–0.096
*a_p_*_1_ (mm)	0.80–1.20	*a_p_*_3_ (mm)	0.08–0.12
*a_e_*_1_ (mm)	0.40–0.60	*ν_T_* (mm/s)	200–400

**Table 9:** Comparison of equipment process parameters before and after optimization.

Process Parameter	Value		Process Parameter	Value	
	Before optimization	After optimization		Before optimization	After optimization
*n*_1_ (r/min)	1200	1194	*n*_3_ (r/min)	960	960
*f*_1_ (mm/r)	0.100	0.108	*f*_3_ (mm/r)	0.080	0.090
*a_p_*_1_ (mm)	1.2	1.2	*a_p_*_3_ (mm)	0.12	0.12
*a_e_*_1_ (mm)	0.3	0.6	*ν_T_* (mm/s)	250	393

**Table 10:** Objective values before and after process parameter optimization.

Optimization Objective	*I*		*n_R_*		*R_C_*	[*R_l_*, *R_u_*]
	Before optimization	After optimization	Before optimization	After optimization		
Dynamic energy consumption of machine tool M1 (J)	74,877.4	51,076.9	0.45	0.69	120,000	[0, 100,000]
Dynamic energy consumption of machine tool M2 (J)	44,933.4	43,887.1	0.69	0.70	100,000	[0, 80,000]
Dynamic energy consumption of robots R1, R2, and R3 (J)	6096.1	4797.7	0.49	0.65	10,000	[0, 8000]
Number of cutting workpieces within the tool life	16	15	0.55	0.45	0	[10, 21]
Robot maximum power (W)	387.0	509.0	0.75	0.40	1000	[350, 700]
Production time (s)	1098	848	0.40	0.65	1500	[0, 1000]

## Data Availability

The original contributions presented in this study are included in the article; further inquiries can be directed to the corresponding authors.
